# Fever of Unknown Origin From the Primary Care Perspective: A Case Report

**DOI:** 10.1155/carm/9086960

**Published:** 2026-02-27

**Authors:** Kalsang Chodon, Shakir M. Saud, Angela R. Rodgers

**Affiliations:** ^1^ Family Medicine Residency, Contra Costa Health, Martinez, California, USA; ^2^ Department of Adult and Family Medicine, Conta Costa Health, Martinez, California, USA; ^3^ Department of Hospital Medicine, Contra Costa Regional Medical Center, Martinez, California, USA; ^4^ Department of Emergency Medicine, Contra Costa Regional Medical Center, Martinez, California, USA; ^5^ Department of Family and Community Medicine, University of California, San Francisco, California, USA, berkeley.edu

**Keywords:** family medicine, fever of unknown origin, infectious disease, liver abscess, primary care

## Abstract

A 60‐year‐old immunocompetent man presented to his primary care physician with 3 weeks of fever without an obvious source or risk factors. After multiple encounters, he was found to have a pyogenic liver abscess on computed tomography (CT). He improved with interventional radiology (IR) drainage and antibiotics. Primary care and outpatient providers should be prepared to initiate an appropriate workup for fever of unknown origin (FUO). Family medicine providers working in acute care settings such as urgent care and the emergency department similarly need a framework for FUO. Once common causes have been excluded, pyogenic liver abscess should be considered.

## 1. Introduction

Fever of unknown origin (FUO) is commonly reported in pediatrics and internal medicine. However, despite its prevalence of 1%–3% in hospitalized patients, FUO is not commonly discussed in primary care [1, 2]. Family medicine is a diverse specialty with clinicians working within inpatient and outpatient settings; therefore, it is essential to highlight FUO as it is seen in multiple healthcare settings. We describe the case of a middle‐aged African American man with FUO and no clear predisposing risk factors, whose diagnostic journey began in primary care clinic and continued under the care of family medicine physicians in a county emergency department and hospital ward.

## 2. Case Presentation

A 60 year‐old African American man with history of gastritis, prediabetes, and remote history of gunshot wound presented to his primary care clinic for 3 weeks of fever and chills.

The patient fished 3 weeks previously, but denied eating any fish, swimming in fresh water, puncture injury, animal bites, sick contacts, livestock interaction, hiking, recent travel abroad, or intravenous drug use. During multiple urgent care and emergency department visits for fevers, he had a physical exam that was nonlocalizing without diagnostic clues, normal white blood cell (WBC) count, lactate, blood culture, urine culture, and chest radiography (except for old subsegmental atelectasis). He was discharged as probable viral illness with acetaminophen, but despite antipyretic use, his fever persisted.

The patient was sent back to the emergency department for persistent fever. In addition, he endorsed decreased appetite, weight loss, abdominal pain, and an episode of nonbloody diarrhea. His pulse was 110 beats per minute (bpm) with increased work of breathing. On exam, dental cavities were noted, and lungs were clear on auscultation with regular heart sounds without murmurs. Abdomen was slightly distended with hypoactive bowel sounds, but soft and nontender to palpation. There were no rashes or joint swelling. Laboratory results showed elevated WBCs of 16.7 TH/uL, a normochromic normocytic anemia with hemoglobin of 10.6 g/dL, a C‐reactive protein of greater than 40 mg/dL, an acute kidney injury with a creatinine of 1.5 mg/dL (baseline 0.9 mg/dL), and a normal urinalysis. Testing for HIV, hepatitis C, hypothyroidism, and blood and urine cultures were negative. Stool for ova, parasites, amoebic enzyme immunoassay (EIA), *Clostridium difficile*, *Campylobacter jejuni*, and cultures were negative.

**FIGURE 1:**
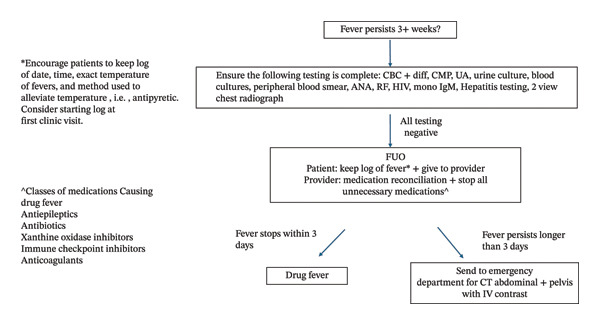
Fever of unknown origin recommendations for primary care providers. Adapted from O. Mourad et al., A Comprehensive Evidence‐Based Approach to Fever of Unknown Origin, https://jamanetwork.com/journals/jamainternalmedicine/fullarticle/215227. FUO: fever of unknown origin; CBC + diff: complete blood count + differential; CMP: comprehensive metabolic panel; UA: urinalysis; ANA: antinuclear antibody; RF: rheumatoid factor; Mono: mononucleosis; CT: computed tomography scan; IV: intravenous.

Computed tomography (CT) revealed a multicystic structure in the liver measuring 5.5 × 4.7 × 6.0 cm (cm), the largest cyst measuring 3.3 × 4.1 × 3.4 cm, and small cystic foci within both lobes of the liver with a thrombosed right hepatic vein suggestive of hepatic abscess with microabscesses or less likely cystic neoplasm with satellite lesions (Figure 1). Echo demonstrated a normal ejection fraction without valvular vegetations. Intravenous ceftriaxone 1 gm q24 h and metronidazole 500 mg q8 h was initiated.

Drainage of purulent fluid from the liver abscesses was performed by interventional radiology (IR). The patient’s shortness of breath resolved after drainage, most likely due to subbdiaphragmatic irritation from the abscesses. The IR fluid showed neutrophils, but was negative for ova, parasites, or bacteria growth.

Initial differential diagnoses included pyogenic infection from biliary or colonic source versus amoebic, but the source of the liver abscess remained unclear, as there was no evidence of diverticulitis, cholecystitis, choledocholithiasis, or cholangitis, although cholelithiasis was found. Liver function tests remained normal. With continued antibiotics, his symptoms improved. He was discharged with oral cefdinir and metronidazole for 4 weeks. On repeat CT, there was size decrease of the liver abscess. Outpatient upper and lower endoscopies were only notable for colonic diverticulosis.

## 3. Discussion

FUO poises a significant diagnostic challenge, particularly in primary care. This case of a 60 year‐old immunocompetent man without obvious risk factors with a pyogenic liver abscess presenting as FUO, underscores the importance of a high index of suspicion and systemic diagnostic approach for patients with persistent fevers. While pyogenic liver abscesses are a rare cause of FUO, it is an important differential as delayed diagnosis can result in abscess rupture, peritonitis, and life‐threatening sepsis. In this case, early imaging and intervention likely contributed to a timely recovery. Notably, no organism was identified on cultures; however, significant improvement was seen on interval imaging with continued antibiotics despite nondiagnostic testing.

**FIGURE 2:**
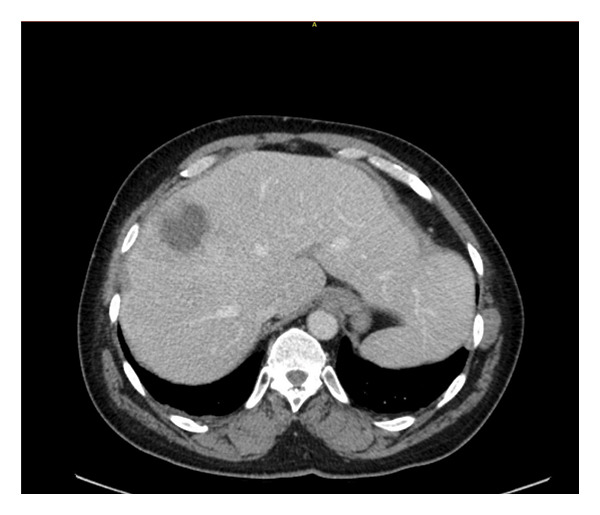
Cross‐sectional view of computed tomography (CT) demonstrating hepatic abscess (white arrow) in the right lobe.

The definition of FUO has had several iterations [3]. Despite various criteria and recommended diagnostic testing, the 3‐week fever duration without explanation has been consistent, as benign febrile illnesses are self‐limiting [4]. The 2021 FUO criteria described by Wright et al. define FUO as a fever of 38.0°C (100.4°F) or more for at least 3 weeks in an immunocompetent patient without an apparent diagnosis after a standard minimal diagnostic protocol [4]. A fever beyond 3 weeks warrants a fever log, discontinuation of nonessential medication, basic testing, and expedited imaging (Figure 2).

Major causes of FUO include infectious, noninfectious, malignancy, or miscellaneous, although 25% have no identifiable source [5]. Pyogenic liver abscesses are a rare cause of FUO and often occur in patients with biliary tract disease and intraabdominal and/or systemic infection. The incidence of liver abscesses is 2.3 cases per 100,000 people, with a male predominance. A significant percentage of pyogenic cases are associated with cholangitis [6]. Hematogenous spread from endocarditis, parasitic infections, trauma, or malignancy are other causes [7]. A notable finding in this case was the presence of multiple dental caries. Increasing evidence links poor dentition and odontogenic disease to pyogenic liver abscesses, particularly in cryptogenic cases. A population‐based study by Lai et al. demonstrated an association between periodontal disease and pyogenic liver abscesses [8, 9]. In addition, several case reports have identified oral flora, including *Streptococcus anginosus* and anaerobes, from cultures in patients with pyogenic liver abscesses [9]. Although cultures were negative, odontogenic disease remains a possible hematogenous source.

CT with contrast is the preferred imaging for diagnosis with procedural aspiration for abscesses less than 5 cm or catheter placement if larger than 5 cm [10]. Aspirate is sent for Gram stain and culture to guide treatment. Empiric antibiotic therapy is initiated prior to diagnosis and should cover for Gram‐positive, Gram‐negative, and anaerobic organisms pending culture results. A standard combination is a third‐generation cephalosporin and metronidazole.

In summary, this case adds to the literature describing cryptogenic pyogenic liver abscess as an uncommon but clinically significant cause of FUO and highlights the importance of considering dental pathology as a potential infectious source. Early imaging, recognition of atypical risk factors, and prompt treatment are essential to preventing serious complications. This case underscores the need to include pyogenic liver abscess in the differential diagnosis of FUO, even in the absence of clear predisposing conditions, and demonstrates the critical role of timely imaging in establishing the diagnosis. As primary care and family medicine clinicians increasingly manage complex patients across care settings, familiarity with the evaluation and management of FUO is essential to improving diagnostic accuracy and patient outcomes.

## Funding

There was no external funding required for the completion of this case report. So, no funding was received for this study.

## Conflicts of Interest

The authors declare no conflicts of interest.

## Data Availability

The data supporting this study′s findings are available from the corresponding author upon request.
